# A Conservative Restorative Protocol for Symptomatic Irreversible Pulpitis: Clinical Success and Rapid Pain Relief in a 27-Case Series with 36-Month Follow-Up

**DOI:** 10.3390/dj14090571

**Published:** 2026-09-07

**Authors:** Zachary Zhaoqian Pan, Thomas Pan

**Affiliations:** 1 Private Practice, Provincial Dental, 1955 Provincial Road, Windsor, ON N8W 5V7, Canada; 2 Department of Physiology and Pharmacology, University of Western Ontario, London, ON N6A 3K7, Canada; tpan47@uwo.ca

**Keywords:** symptomatic irreversible pulpitis, post-operative pain, solid glutaraldehyde, Pulpitis Restorative Protocol (PRP), vital pulp therapy (VPT), reversible pulpitis

## Abstract

**Background/Objectives**: Symptomatic irreversible pulpitis (SIP) is conventionally treated with pulp tissue removal via root canal treatment, pulpectomy, or pulpotomy. This case series introduces the Pulpitis Restorative Protocol (PRP), a novel method used to treat SIP without pulp removal, relieve pain, and maintain a positive pulp sensibility response. **Methods**: In this retrospective case series (March 2022–March 2025), 17 SIP patients (plus 10 reversible pulpitis [RP] control cases) were treated with the PRP utilizing a bioactive glutaraldehyde (GA)-resin-modified glass ionomer (RMGI) solid matrix. Follow-up to 36 months (average 17.37 ± 9.61 months) included clinical examination, cold sensibility testing, and radiographs. **Results**: Treatment was successful in 15/17 SIP cases, an 88.2% success rate, and 9/10 RP cases, a 90.0% success rate. Overall, a total of 24 of 27 cases were successful, a rate of 88.89%. Success criteria were the absence of pain, no soft-tissue abnormality, a positive cold test response, and no radiographic apical lesions. No failures occurred in the first year; two cases failed in the second year and one in the third year. The PRP provided favorable pain relief: 74.1% of patients reported minimal post-operative pain (0–1/10 VAS) the evening of treatment, and 96.3% of patients reported no pain at 24 h without analgesics. All 24 successful cases maintained a positive pulp sensibility response at their final visit. Radiographs were stable in 24 cases. In vitro *experiments showed* that the GA-RMGI solid matrix exhibits contact-independent bactericidal activity. **Conclusions**: The PRP provides a promising approach to SIP management, offering a conservative, cost-effective, and efficient alternative to conventional endodontic treatments alongside favorable pain relief. Its clinical significance warrants further verification in various clinical settings and randomized clinical trials.

## 1. Introduction

Symptomatic irreversible pulpitis (SIP) is most commonly caused by deep caries and is characterized by symptoms of spontaneous, constant, and severe pain, which profoundly impact quality of life [1,2]. The definitive standard of care is root canal treatment (RCT), which aims to preserve the affected tooth [3]. However, RCT is a complicated, time-consuming, and costly procedure that weakens tooth structure, sacrifices tooth vitality, and may lead to post-operative discomfort [1,2]. It also renders the tooth more susceptible to fracture [4,5]. Furthermore, RCT-treated teeth often require subsequent crown restoration, further complicating care and escalating the financial burden for many patients [4,5,6,7]. Multiple factors, including tooth-related anatomical complexity and the operator’s training and competence, also influence the long-term success of RCT [8,9,10,11].

In response to these limitations, interest has grown in vital pulp therapies (VPTs) as tooth-preserving alternatives [12,13,14,15]. Some studies have found that full pulpotomy for SIP has success rates similar to those for RCT [16,17,18]. While less invasive than RCT, partial and complete pulpotomy remain constrained by the need for pulp exposure, strict aseptic technique, and subjective intraoperative assessment of bleeding and tissue appearance [19]. These requirements underscore the technique-sensitive and operator-dependent nature of pulpotomy, particularly in emergency settings where general dentists are often the first-line providers. Critically, a key patient-centered outcome and the sole motivation for emergency dental visits for most SIP patients—immediate and reliable pain relief—remains a challenge for both RCT and pulpotomy [20,21,22]. A significant proportion of patients require analgesic, even narcotic medication after treatment, with some needing additional emergency care for pain within a week [23,24,25,26].

The historical limitation of these therapeutic options stems from the traditional clinical diagnosis of SIP, which is based on the “strangulation theory.” This theory suggests that the increasing intrapulpal pressure caused by pulpitis within the rigid dentinal chamber unavoidably leads to localized ischemia, widespread necrosis, and a total loss of healing capacity [27]. Despite recent research exposing the limitations of this classic diagnosis, current treatment options remain invasive [28,29].

Thus, an ideal treatment for SIP would be conservative, simple to perform by general dentists, cost-effective for patients, and capable of both preserving pulp sensibility and ensuring reliable and rapid symptom resolution without reliance on medication. This case series introduces and evaluates a novel, non-invasive pulp-preserving technique utilizing bioactive solid glutaraldehyde (GA). We present a retrospective case series of 27 mature permanent teeth with SIP or reversible pulpitis (RP) treated with this protocol, which we name the Pulpitis Restorative Protocol (PRP), and followed for up to 36 months, assessing its potential as a new pathway in minimally invasive SIP management.

## 2. Materials and Methods

### 2.1. Case Series Overview and Case Selection Criteria

From March 2022 to March 2025, a single clinician (Z.P.) provided PRP treatment to 33 patients in a private dental office setting. These patients, who were treated at different times during that period, were selected based on the specific indications for PRP therapy (Table 1). At the initial appointment, a comprehensive medical and dental history was obtained alongside a thorough clinical examination. The medical histories included immunocompromising conditions and medications. The dental history evaluated prior trauma, recent restoration, and detailed pain characteristics, including pain onset, duration, location, quality and triggering and exacerbating factors, with specific note of recurrent, severe and spontaneous episodes of the same tooth. Associated symptoms including soft tissue swelling or discharge were recorded. Pain severity was quantified using a 0–10 Visual Analog Scale (VAS) for both maximum pain and pain at time of assessment.

An extra-oral examination assessed facial soft tissue, the temporomandibular joints (TMJs), and the sinuses. An intra-oral examination documented caries, restorations, fractures, mobility, periodontal status, and occlusion. Diagnostic tests included percussion, palpation, and a standardized cold sensibility test (CST). For the CST, the tooth was isolated and dried. After testing control teeth, the suspect tooth was stimulated on the buccal or lingual surface with Endo-Ice (COLTENE, Cuyahoga Falls, OH, USA) on a cotton Q-tip for ≤15 s or until response. Periapical and bitewing radiographs were obtained.

After examination, diagnosis, treatment plan, and treatment options were presented to the patients (or parents). Diagnosis of SIP cases in this study was based on the following signs and symptoms, which represent clinically practical thresholds derived from contemporary diagnostic terminology: (1) spontaneous and constant pain persisting > 1 h; (2) severe pain (≥5/10 VAS); (3) presence of STP; and (4) pain and lingering pain (≥10 s at CST). These diagnostic thresholds were chosen to identify teeth with clinically significant pulpal inflammation while excluding teeth with necrotic pulps or apical pathology. It should be noted that the clinical diagnosis of SIP is a symptom-based category and does not directly establish the histopathological status of the pulp, including the presence, location, or extent of inflammation or necrosis. All cases diagnosed as SIP met all four criteria except for one case, who reported pain at 4/10 on the VAS but met the other three criteria. One case reported pain at 5 on the VAS but lacked spontaneous pain, and pain at CST was diagnosed as RP. An electric pulp test was not used because it was found to be unreliable on control teeth when initially used in some cases.

For patients diagnosed with SIP, treatment options—including dental extraction, root canal treatment, pulpotomy (whether by a specialist or performed in-office), and PRP therapy—were thoroughly explained. These discussions included the associated risks and benefits of each option. For PRP therapy, additional information was provided, including its uncertain success rate and long-term prognosis, alternative treatment plans in the event of failure, and associated costs. After all questions were addressed, patients made informed decisions regarding their care. Only patients who elected for a conservative approach and provided specific informed consent for PRP therapy were provided with the PRP treatment protocol.

All the teeth included in this case series presented with deep caries, except for two teeth with compromised restorations. At the diagnostic examination, all selected teeth demonstrated signs and symptoms ranging from RP to SIP, exhibited no periapical radiographic lesions, and yielded a positive response to CST (ranging from normal sensation to extreme pain/or lingering pain). Exclusion criteria comprised a negative response to CST or radiographic evidence of apical pathology (e.g., apical lucency, widening of the periodontal ligament space, or loss of the lamina dura) or associated soft tissue abnormalities such as swelling, sinus tracts, or fistulae.

### 2.2. PRP Procedures

#### 2.2.1. Cavity Preparation and Selective Caries Removal Protocol

The cavities were prepared by removing carious enamel and peripheral dentin to establish an adequate restorative form under local anesthesia using a high-speed diamond bur with water spray cooling, following the selective caries removal method. The remaining carious tissues from the cavity’s peripheral walls were removed using a slow-speed round carbide bur, followed by a hand spoon excavator, until sound enamel and firm dentin were reached. This procedure ensured a peripheral seal zone of approximately 1–2 mm for composite resin bonding. When pre-operative intraoral radiographs indicated a cavity dentin thickness of ≤1.0 mm over the pulp horns or chamber, the remaining caries were selectively removed meticulously using only a hand spoon excavator. Soft dentin was left in situ in instances of doubt to avoid potential pulp exposure.

#### 2.2.2. Modified Sodium Hypochlorite Treatment and GA-RMGI Solid Matrix Formation

Upon completing the preparation, we gently packed the cavity with a cotton pellet saturated with buffered sodium hypochlorite (pH8.2, B-NaOCl) for at least 3 min. The cavities were rinsed with water after the removal of the cotton pellet and then etched with 37% phosphoric acid for 30 s on enamel and 15 s on dentine. The cavities were rinsed for 10 s with water, and the cavity was briefly air-dried with an air syringe (acid etching was omitted when using amalgam restorations). We then rinsed the cavity again with the same B-NaOCI and briefly air-dried the cavity. Next, a coat of liquid GA (5–10% GA, 25–50% 2-hydroxyethyl methacrylate HEMA) was applied to the dentine surface of the cavity floor and walls with a medium-sized micro-applicator saturated with ~10 µL of GA (Figure S1). The dentin surface of the cavities should appear glossy and wet, but no pooling occurred (~5 µL GA left on cavity surfaces). Then, we mixed light-cured RMGI and loaded it over to liquid GA on the dentine surface to form an even thickness of~1.0 mm of the GA-RMGI mixture with a Dycal applicator (Dentsply Sirona, Charlotte, NC, USA). The GA-RMGI mixture was cured for 20 s with a 1200 W/cm^2^ intensity LED curing light. Then, a coat of dental adhesive material was applied to the entire cavity floor and wall surfaces, including the GA-RMGI surface and enamel with a new micro-applicator, followed by 5 s of curing with the same curing light. A thin layer of flowable composite was applied as an intermediate sealing and adaptation layer over the GA-RMGI matrix. It was intended to improve cavity-floor adaptation, reduce interfacial voids and potential microleakage, and provide stress absorption during polymerization. In the PRP, it also served to seal and protect the GA-RMGI matrix from potential GA vapor escape and mechanical disruption during placement of the definitive restoration, especially amalgam condensation, while providing additional thermal insulation when amalgam was used. The necessity and relative contribution of these functions require further clinical investigation [30,31].

#### 2.2.3. Placement of Final Restorative Material of Composite Resin or Amalgam

Following light-curing of the flowable composite, the remaining cavity was restored using either amalgam or composite resin of various brands as conventional restorations. Final restorations consisted of twenty-four composite resin and three amalgam restorations. The occlusion was checked, adjusted, and polished. Patients were advised to avoid whitening products such as whitening toothpaste and extreme temperatures in beverages and food for two weeks. During the follow-up period, no patients reported using tooth whitening products; however, six patients used toothpastes containing whitening chemicals unintentionally and found out afterwards. No analgesics or antibiotics were routinely prescribed; however, patients were advised that if they felt pain after local anesthesia subsided, they could take the over-the-counter ibuprofen or acetaminophen that evening and the following morning. The patients were informed that mild post-operative pain should completely disappear at 24 h.

### 2.3. In Vitro Bactericidal Experiment

A preliminary in vitro bactericidal assay was performed to assess the contact-dependent and contact-independent antimicrobial activity of GA-containing formulations; the detailed experimental procedures are provided in the Supplementary Materials.

## 3. Results

### 3.1. Patient Demographics and Treatment Outcomes

This report included only patients who underwent a post-operative clinical examination at least 3 months after PRP treatment, as assessment at an earlier time point may not provide a sufficiently reliable indication of treatment success. However, this follow-up requirement introduces a potential source of selection and attrition bias, as patients who experienced treatment failure may have been less likely to return for clinical reassessment. Conversely, patients who experienced successful treatment and complete resolution of symptoms may also have been less likely to return for an in-office recall examination, particularly if they generally sought dental care only when symptoms became severe. Six patients were therefore excluded from the final analysis because they did not return for post-operative clinical examination or could not be contacted for further follow-up. Two of these six patients had permanently returned to their home countries. Nevertheless, during their last post-operative telephone interviews, all six patients reported no residual pain and indicated that they were satisfied with the treatment outcome. These factors should be considered when interpreting the treatment success rate and the generalizability of the findings. A sensitivity analysis was performed: if all six lost-to-follow-up patients are considered failures, the success rate is 24/33 (72.7%); if all are considered successes, the success rate is 30/33 (90.9%). The reported success rate of 24/27 (88.9%) is the complete-case estimate; sensitivity analysis yielded a range of 72.7% to 90.9%. This retrospective case series gathered data from twenty-seven patients (17 males, 10 females) with a mean age of 38.2 ± 14.7 years (median: 38 years, range: 15–71). Based on the initial examination, 17 patients were diagnosed with SIP and 10 patients with RP (Table 2). The average follow-up duration was 17.37 ± 9.61 months. Treatment success standards included all of the following four criteria: (1) absence of pain; (2) absence of soft tissue abnormalities; (3) absence of radiographic lesion; and (4) positive response to cold sensibility test.

Overall, 24 out of 27 cases were symptom-free and maintained a positive pulp sensibility response at their last dental recall exam, yielding an overall success rate of 88.9%. Specifically, nine out of ten RP cases were successful (90.0%), and fifteen out of seventeen SIP cases were successful (88.2%). This study included eighteen molars and nine premolars. Notably, no anterior teeth with deep carious lesions and concomitant SIP or RP were encountered during this period.

Approximately one-third of patients experienced post-operative temperature sensitivity at various time points after PRP treatment. The treatment had a similar post-operative sensitivity rate to that of conventional composite resin restoration [32]. These cases were mostly SIP cases, multi-surface cavities, or cavities with sub-cemento-enamel junction (CEJ) margins. Post-operative sensitivity rarely occurred with class I restorations and with amalgam restorations. This sensitivity was alleviated by one or more of the following methods: avoiding extremely hot or cold food and drinks; discontinuing whitening products (such as whitening strips or whitening toothpaste and mouthwash); applying a coat of GA followed by a thin layer of RMGI to the restoration margin, then light curing; or redoing the defective or fractured restorations.

### 3.2. Pain Reduction

Post-operative pain was assessed using a 0–10 VAS at three time points: the evening of treatment (day 0), 24 h post-operation, and 48 h post-operation. Data collection was conducted via telephone. During the 24 h follow-up call, most patients provided two separate scores: a retrospective recall of their day 0 evening pain and their current 24 h pain score. A minority of patients were successfully contacted and scored on the actual evening of their treatment. All 24 h and 48 h follow-up calls were pre-arranged with patients in the office immediately after their procedure, utilizing a combination of inbound patient calls and outbound doctor calls.

Post-operatively, 74.1% of patients reported minimal or no pain (VAS 0–1/10) the same evening of PRP treatment, with 96.3% achieving pain-free status without analgesics by 24 h. Success in post-operative pain reduction was defined as a VAS score of ≤1/10. No analgesics or antibiotics were routinely prescribed. We observed a strong, positive correlation between pre-operative pain and post-treatment reduction in patients with pre-operative spontaneous pain (Mann–Whitney U, *p* < 0.001) (Table 3 and Table 4). Thus, while patients with high initial pain benefited from the greatest absolute decrease, they typically retained some residual pain due to their elevated baseline, whereas those with milder pre-operative pain were more frequently pain-free on the evening of treatment. By comparison of different subgroups for their immediate pain reduction in the same evening, there is no difference between sexes. However, when clustering the cases into two groups according to median age (age ≤ 38 years and age > 38 years), the younger group had far better immediate pain reduction than the older group the evening of the treatment (*p* = 0.019) (see Table 4).

### 3.3. Post-Operative Radiographic Changes

**Figure 1:**
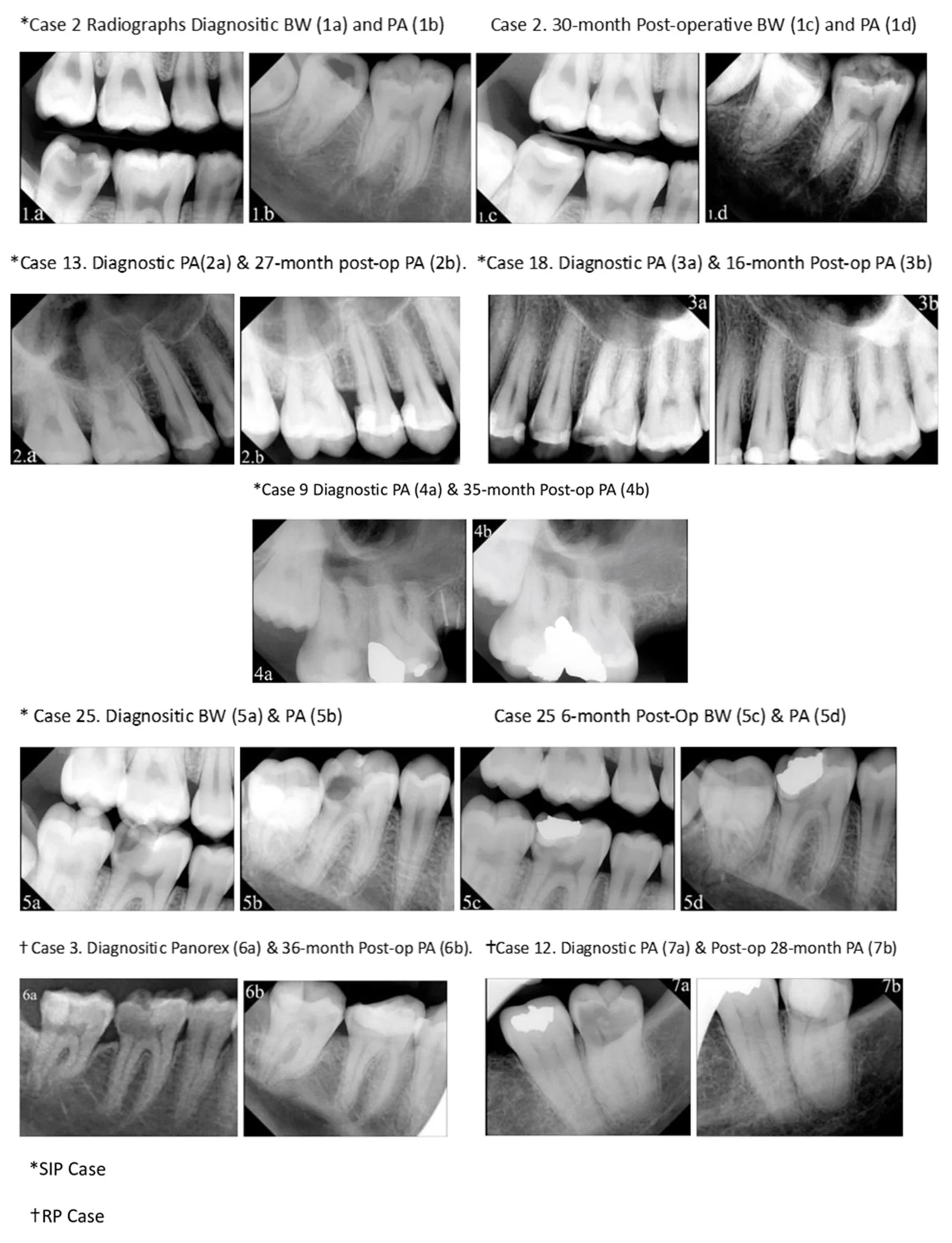
Selected case radiographs.

There was no post-operative radiographic change in twenty-four cases, and the radiographic success rate was 88.9% (Figure 1). Three failed cases had onsets of recurrent pain after 17 months, 23 months, and 31 months of symptom-free post-operative period, respectively, and required RCT. The demineralized affected dentin left in situ during cavity preparation maintained a stable radiodensity, with no evidence of remineralization. Furthermore, no tertiary dentin formation was observed on any post-operative radiographs. Radiographic changes did not consistently correlate with clinical outcomes. Of the three failed cases, two showed no radiographic alterations. Conversely, two successful cases (asymptomatic with clinical findings consistent with a vital pulp) exhibited atypical radiographic findings, characterized by well-defined balloon-shaped radiopaque borders around the apex with no internal density changes. Both teeth received a second PRP treatment due to lengthy sensitivity to cold stimulation that recurred months after the first restoration. In one failed SIP case, symptoms of cold sensitivity returned 18 months post-PRP treatment, and radiographs revealed a defect restoration margin located apical to the CEJ. We recommended replacement of the defective restoration, but the patient declined. At the 24-month follow-up, the tooth presented with spontaneous pain and typical apical radiolucency. The etiology of the failure in the remaining two cases could not be identified. The PRP case data and their treatment results are listed in Table S1, and some selected case radiographs are presented below.

### 3.4. In Vitro Bactericidal Experiment Result

The GA–RMGI matrix demonstrated contact-independent antimicrobial activity in the sealed-dish assay, whereas comparable activity was not observed for GA–TheraCal LC or GA–Lime-Lite™ Enhanced (Supplementary Figures S2–S4 and Table S2).

## 4. Discussion

The long-term clinical and radiographic stability observed in this 36-month case series challenges the traditional view of pulpal irreversibility. Our findings provide clinical evidence that teeth clinically diagnosed with SIP can recover without endodontic intervention. Although Ricucci et al. found that clinical and histologic classification of pulp conditions revealed good agreement [33], it should be emphasized that the clinical diagnosis of SIP is a symptom-based category and does not directly establish the histopathological status of the pulp, including the presence, location, or extent of inflammation or necrosis. This recovery may be achievable if the microbial load is effectively eliminated and the local tissue environment is biochemically restored before irreversible microvascular collapse occurs.

To achieve this SIP recovery, we present a novel, non-endodontic SIP therapeutic alternative, the PRP, which innovatively uses the biochemical properties of GA. GA is a five-carbon dialdehyde that forms stable cross-links with protein functional groups, including amines, thiols, phenols, and imidazoles [34,35]. While GA has been used almost exclusively in liquid form, including in pulpotomy of primary teeth and dentine desensitization, our PRP delivers GA as a solid matrix formed in situ. This solid system has three advantages over liquid-form GA, as discussed later: it reduces pulp inflammation, achieves profound and rapid post-operative pain relief, and preserves pulp sensibility. Ultimately, our 36-month clinical success supports the concept of using next-generation biomaterials for pulp tissue immunomodulation [36].

Clinically, dentists are familiar with the PRP because it is similar to indirect pulp capping (IPC); however, the PRP serves as a distinct clinical function compared to IPC. IPC acts as passive pulp protection, while the PRP may promote active pulp healing and preservation. This functional transition is thought to be achieved through three synergistic innovations designed to harness GA’s therapeutic potential while mitigating its inherent cytotoxicity: (1) formation of a solid GA matrix; (2) creation of an ideal substrate pH; and (3) delivery of a precise therapeutic dosage.

### 4.1. Development of a Solid Bioactive GA Matrix for Multi-Mechanistic Therapy

A primary innovation is the in situ formation of a solid GA–RMGI bioactive matrix by applying a layer of RMGI liner directly over liquid GA on the cavity floor/walls. The two components integrate readily, likely because both contain HEMA. Subsequently, the GA–RMGI mixture is light-cured to form a cohesive solid matrix. This solid formulation overcomes several critical limitations associated with traditional liquid GA alone, including rapid, uncontrolled self-polymerization [37], non-productive binding to adjacent proteins in direct contact with GA liquid [34,35], poor diffusion through dentin [38,39], and potential cytotoxicity associated with GA-containing formulations [40].

The conversion of liquid GA into a GA–RMGI solid matrix may help localize the material within the prepared cavity while reducing premature GA self-polymerization and non-productive binding, thereby preserving reactive aldehyde groups. Crucially, this solid matrix may also allow for the release of GA vapor monomers. Because GA is a small dialdehyde molecule (molecular weight 100.12 g/mol) with relatively high vapor pressure [41], these volatile GA monomers may diffuse through the dentinal tubule network [34,35,42,43] and reach areas not in direct physical contact with the GA–RMGI solid matrix. This is a potential advantage in minimally invasive vital pulp therapy. This diffusion is facilitated by the hydrophilic nature of dentin. Studies have shown that GA can diffuse through dentin, although slowly, and that this diffusion is concentration-dependent and time-dependent [34,35,38]. Notably, GA in its vapor phase can permeate and cross-link both surface-exposed as well as internal amine groups of microbial and host proteins [42]. However, whether GA or specific volatile GA species released from the cured matrix actually diffuse through dentinal tubules under clinical conditions remains to be established.

The potential for contact-independent antimicrobial activity was explored in preliminary in vitro experiments. The complete experimental methods and results are provided in the Supplementary Materials (Figures S2–S4 and Table S2). Briefly, direct contact of oral bacterial cultures to GA/HEMA or Gluma Desensitizer (Kulzer, Wehrheim, Gemany) produced minimal inhibition (<1 mm), whereas indirect exposure in sealed Petri dishes without direct agar contact produced larger inhibition zones of approximately 6–7.5 mm and reduced colony counts. The GA–RMGI solid matrix demonstrated measurable bactericidal activity in the absence of direct contact, with the greatest effect observed at approximately 4 h, diminishing by 12 h and becoming undetectable by 24 h. In contrast, GA–TheraCal LC and GA–Lime-Lite™ Enhanced formed solid matrixes that did not demonstrate comparable contact-independent activity, suggesting that this property may depend on the specific GA–RMGI formulation. These findings provide preliminary evidence consistent with contact-independent antimicrobial activity of the GA–RMGI matrix under the experimental conditions. They may not, however, establish bacterial killing in vivo, GA diffusion through dentinal tubules, clinical efficacy, or safety. The greater activity observed during indirect exposure may reflect differences in GA availability and consumption within the experimental system, but this interpretation remains speculative and requires further investigation.

The proposed contact-independent activity is also consistent with the known protein-reactive properties of GA. GA can interact with amine-containing groups in proteins and produce covalent cross-linking [42]. Scheffel et al. found that when GA is used with HEMA, as in most dental uses (Gluma Desensitizer), it is cytotoxic; however, if GA is used alone, it is not toxic to odontoblast-like cells [40]. Within the PRP approach, light curing is intended to polymerize and immobilize the HEMA-containing RMGI component while retaining GA within the solid matrix. This formulation may therefore provide a means of localizing the GA-containing material while potentially permitting the release of volatile GA species. Nevertheless, the relative contribution of vapor-phase GA, residual soluble GA, or other formulation-specific factors to the observed antimicrobial activity remains uncertain and cannot be resolved by the present experiments.

From a pathophysiological perspective, the progression of SIP involves a cascade of events that the PRP is hypothesized to interrupt. Bacterial invasion through deep caries triggers odontoblast and immune cell activation, releasing inflammatory mediators (including interleukins, prostaglandins, and neuropeptides such as substance P and CGRP) that cause vasodilation, increased vascular permeability, and tissue edema [44,45,46]. Within the low-compliance environment of the rigid dentinal chamber, this inflammatory exudate increases intrapulpal pressure, which can compress thin-walled venules, reduce venous outflow, and create localized tissue acidosis and hypoxia [47,48]. The resulting acidotic environment is both cytotoxic to pulp stem cells and a direct stimulant of nociceptors, contributing to the characteristic severe pain [49,50].

The PRP is hypothesized to interrupt this pathophysiological cycle at multiple points: (1) by reducing the microbial and toxin burden through GA-mediated cross-linking of bacterial proteins [43,51]; (2) by conditioning the acidic cavity environment with B-NaOCl, which buffers the superficial dentin pH and establishes favorable conditions for odontoblast survival and GA activity [37,52,53]; (3) by deactivating inflammatory mediators through non-specific covalent cross-linking [35,54,55]; and (4) by occluding dentinal tubules through protein coagulation, reducing hydrodynamic sensitivity [56,57]. These combined effects may reduce nociceptor stimulation and allow residual vascular pulp tissue to recover before irreversible microvascular collapse occurs. However, this mechanistic framework remains hypothetical and requires validation through histological studies and vascular perfusion assessment.

### 4.2. The Clinical Activation of GA Biocidal Activity: Resolving the pH Dilemma

GA possesses potent biocidal and protein-crosslinking properties, but its efficacy is critically constrained by a narrow optimal pH window of 7.5–8.5 [37,58]. This presents a fundamental clinical challenge because commercially supplied GA for dentists is formulated in acidic solutions (e.g., Gluma Desensitizer pH3–4) to ensure shelf-life stability, inherently suppressing GA activity upon application [37,59]. GA is largely ineffective in acidic environments or at highly alkaline extremes, with peak activity occurring only at neutral to weak alkaline pH [43].

The PRP addresses this dilemma by buffering target tissue rather than directly buffering the GA solution itself. The keystone of this approach is frequent irrigation of the cavity with pH-buffered sodium hypochlorite (B-NaOCl, pH ~8.2) to condition the cavity, followed by placing a B-NaOCl-saturated cotton pellet in the prepared cavity for ≥ 3 min, which elevates the pH of the acidic cavity and the adjacent coronal pulp chamber to a near-neutral range [53]. This single step accomplishes multiple synergistic functions: it establishes the optimal neutral to weak alkaline microenvironment for GA, provides supplemental disinfection, controls hemorrhage, and, critically, neutralizes the tissue acidosis characteristic of inflamed pulp. This acidosis is both cytotoxic to vital pulp stem cells and a direct stimulant of nociceptors, contributing to pain [49,50].

This pre-conditioning of cavity pH is especially crucial when using solid GA, which acts via vapor-phase monomer diffusion. Because GA monomers in the vapor phase do not carry a pH themselves, their ultimate biochemical activity depends entirely on the microenvironment of the remote site they reach. If these vapors encounter an unbuffered, acidic pulp chamber, their biocidal and cross-linking potential is negated. The mechanistic rationale is clear: at weak alkaline pH, microbial surfaces present more binding sites for GA’s irreversible cross-linking action [43]. Therefore, applying commercial acidic GA formulations, or pairing them with regular high-pH (~11) NaOCl, fails to harness GA biocide activity. The PRP transforms the target tissue into an activating platform for GA. This strategy is potentially advantageous compared to buffering the GA solution itself directly.

### 4.3. Optimized Dosage and the Safety of GA in PRP Application

Historically, GA used in dentistry as a pulpotomy fixative or dentinal desensitizer was usually an imprecise liquid application with uncontrolled dosage and unknown retention [60,61,62,63,64,65]. The cytotoxicity of GA is concentration-dependent and time-dependent [40,66,67]. When combined with HEMA in commercial dental materials, GA demonstrated marked toxicity toward dental pulp stem cells because HEMA induces reactive oxygen species and increases permeability [68]. Conversely, isolated transdentinal application of GA alone was not toxic to odontoblast-like cells [40].

The PRP explicitly harnesses GA’s self-limiting chemical nature to utilize GA’s therapeutic effect and to reduce cytotoxic side effects in SIP management. Under the optimal conditions established by the PRP, GA undergoes progressive protein cross-linking and self-polymerization until its limited reactive capacity is depleted [43,58]. The PRP used an empirically GA therapeutic dose of approximately 5 µL remaining on the cavity surfaces after micro-applicator placement, with no visible pooling. This dose was chosen to wet the dentin surface, and was immediately mixed up with RMGI and solidified into a GA-RMGI solid matrix, leaving no free liquid GA. This rationale is supported by the concentration-dependent and time-dependent cytotoxicity of GA and its self-limiting cross-linking and polymerization behavior, whereby GA reactive aldehyde groups are progressively depleted through protein binding and self-polymerization [37,40,43,67]. We hypothesize that the optimal GA dose may vary and depend on cavity surface area, tooth size, the thickness of the dentine layer left between the cavity and the pulp chamber, the age of the tooth, and pulpal inflammation severity. However, the therapeutic dose range remains unsystematically established, necessitating future dose-finding studies. This system ensures PRP safety and efficacy through two mechanisms. The first is physical confinement and differential volatility. Both GA and HEMA are confined to a solid matrix sealed within a restored cavity, limiting the interaction of GA and HEMA with non-target tissue. HEMA monomers are polymerized and trapped inside the solid matrix after light curing. If any unpolymerized HEMA monomers remain after light curing, they likely remain within the solid due to their extremely low vapor pressure. Meanwhile, GA exhibits relatively higher vapor pressure, allowing GA monomers to slowly volatize out of the solid matrix. The second mechanism is a precise dosage for controlled, self-limited biocide activity, where limited GA active binding sites are entirely exhausted within ~24 h, leaving no residual chemically active monomer to cause further long-term tissue injury.

Clinically, this precise dosage resulted in a high success rate of symptom relief and pulp preservation without adverse effects in our study. The post-operative sensitivity rate was similar to that of routine composite restorations but occurred later, typically associated with large, multi-surface subgingival restorations, suggesting that long-term success depends more on final restoration seal than on residual chemical toxicity [30,31,32].

Our in vitro bactericidal assay with a 10 µL GA-formed GA-RMGI solid demonstrated GA’s transient bioactive window, which peaks at 4 h, diminishes by 12 h, and terminates within 24 h (Supplementary Materials, Figures S3 and S4), aligning with findings on its time-dependent cytotoxicity [67].

GA cross-linking procedures are largely developed through empirical observation [34]. The dosage of ≈5 µL GA used in the PRP was also developed empirically for early-stage SIP, where partial necrosis may be limited to the coronal pulp adjacent to the caries [69]. Treatment outcome, however, may be influenced by systemic health. Individuals with immunocompromised conditions, such as cancer or poorly controlled diabetes, may be prone to treatment failure. Conversely, young, healthy patients may often show a better response, as evidenced by their greater immediate pain relief (Table 4). This successful outcome of a young tooth may result from the high vascularity and robust regenerative capacity of young pulp stem cells [70,71].

It remains unknown whether an increased GA dosage would be effective and safe for later-stage SIP when the necrosis involves radicular or even apical pulp. We observed atypical radiographic changes in two re-treatment cases, which remained asymptomatic with clinical findings consistent with a vital pulp. This atypical radiographic change may represent bony tissue response to double GA dosage, which is likely similar to the response of connective tissue to GA, a well-circumscribed, inflammation-free, self-healing lesion observed by Meon in rats [72]. This reinforces that efficacy and safety are optimized for a single, confined application. Further research is required.

### 4.4. Favorable Post-Operative Pain Control

Pain plays a central role in SIP management and is measured as both a pre-operative diagnostic criterion and a treatment success indicator [15]. The PRP produced a distinctly favorable post-operative pain trajectory. Clinically, most of our patients experienced minimal discomfort (VAS 0–1) immediately after treatment, with near-universal resolution to this level at 24 h. All patients, without analgesia, were able to resume their routine activities by the next day, and there were no unscheduled return visits or phone calls regarding persistent pain. This outcome compares favorably to RCT or pulpotomy treatment for SIP, where significant pain often persists for 36–72 h after treatment, and 25% return visits occur within a week due to persistent pain [20,25,26,73].

This rapid analgesia can probably be explained, as above, by the PRP’s multi-target action on the pain pathway. GA directly addresses key etiologic factors: it reduces bacterial load and prevents their direct activation of sensory neurons, deactivates inflammatory mediators, and arrests inflammation, possibly also via apoptosis [43,74,75]. Simultaneously, GA occludes dentinal tubules through protein coagulation, reducing permeability and hydrodynamic sensitivity [65,76]. Concurrently, B-NaOCl provides antimicrobial activity and, upon contact with acidic carious dentin and acidotic inflamed pulp, may shift the local environment toward near-neutral pH. This could reduce acid-mediated nociceptor stimulation while avoiding the extreme alkalinity of conventional NaOCl and potentially improving the biological environment for residual viable pulp cells. Together with the immediate coronal seal, these effects may explain the rapid reduction in post-operative pain.

### 4.5. Clinical Implications and Future Directions

The PRP offers a conservative, biologically driven alternative for early-stage SIP that can be performed by general dentists in a single brief appointment. By emphasizing objective pulp sensibility assessment over traditional pulpitis classification, this novel restorative protocol bridges the gap between conventional restorative procedures and RCT, potentially reducing unnecessary endodontic interventions and preserving pulp sensibility.

Conceptually, the PRP aligns with the biological rationale of VPT in its aim to preserve pulp vitality in the management of SIP; procedurally, however, it differs from established VPT protocols in that it is performed as a purely restorative procedure without intentional pulp exposure or pulpotomy. The three-year Biodentine XP case series by Tosco et al. [77] involved posterior teeth with deep carious lesions and reversible pulpitis—not SIP—and calcium silicate-based materials (MTA, Biodentine) are typically employed in partial or full pulpotomy protocols that require intentional pulp exposure, hemostasis management, and coronal pulp tissue removal [14,17]. In contrast, the PRP represents—to our knowledge—the first clinical report of a purely restorative, no-pulp-amputation protocol applied to teeth diagnosed with SIP. Mechanistically, whereas bioceramic materials are indicated for exposed pulp or pulpotomy procedures, the GA–RMGI matrix is hypothesized to act through glutaraldehyde-mediated protein cross-linking, contact-independent antimicrobial vapor-phase effects, dentinal tubule occlusion, and interruption of inflammatory signaling. Practically, the light-cured RMGI sets in 20 s compared with the 12–15 min initial setting time required for hydraulic calcium silicate cements [77,78], offering distinct advantages for emergency SIP management in general practice. These differences position the PRP as a unique conservative restorative approach rather than a substitute for bioceramic VPT; prospective randomized trials comparing the PRP with conventional RCT and bioceramic partial/full pulpotomy are warranted.

The PRP should be regarded as an alternative to RCT only in carefully selected cases of early-stage SIP where inflammation remains confined to the coronal pulp and the tooth maintains vital status; teeth with advanced pulp necrosis remain indications for conventional endodontic therapy.

This study has several limitations. Its retrospective, preliminary nature, small sample size, absence of a control group, and lack of true pulp vitality testing (e.g., laser Doppler flowmetry or pulse oximetry) constrain the strength of our conclusions. Cold sensibility testing evaluates neural response rather than vascular perfusion; therefore, maintained positive responses should be interpreted as consistent with—but not definitive evidence of—pulp vitality. Additionally, 18% attrition (6 of 33 patients) introduces potential selection and attrition bias. Long-term success relative to standard treatments remains unknown, and the proposed mechanistic hypotheses require validation through future experimental and clinical studies.

Future research should prioritize (1) randomized trials directly comparing the PRP to RCT/pulpotomy in larger patient cases; (2) histological studies to elucidate pulp tissue response to PRP treatment; (3) optimization of dosages for different sizes of teeth, ages of teeth, and stages of SIP, or severity of pulp necrosis; and (4) advanced 3D imaging to understand long-term periapical status and atypical healing patterns.

## 5. Conclusions

Within the limitations of this retrospective case series, the PRP was associated with rapid post-operative pain reduction, stable clinical and radiographic findings, and the maintenance of a positive pulp sensibility response in most followed-up cases. No protocol-related adverse events were observed, but safety and comparative efficacy cannot be established from this uncontrolled design. Randomized controlled trials with standardized follow-up and true pulp vitality testing are required.

## Figures and Tables

**Table 1:** PRP indications and contraindications *.

Indications	Contraindications
Deep caries involving ≥75% thickness of dentine, near or touching pulp on radiographs	Pulp exposure with uncontrollable pulp bleeding
Spontaneous, severe and constant pain up to 24 h	Spontaneous, severe and constant pain longer than 24 h #
Mild to severe pain, waking up from sleep, but the first onset of spontaneous pain on the same tooth	History of repeated spontaneous and severe pain of the same tooth
Positive response to CST, from normal cold sensation to pain and lingering pain	Necrotic pulp, negative response—no cold sensation nor pain to CST
STP but no apical palpation pain	Severe STP associated with apical palpation pain
Normal soft tissue, no swelling and no sinus tract/fistula	Presence of soft tissue swelling or sinus tract/fistula
Normal periapical tissue radiographically	Radiographic evidence of periapical pathology
Restorable tooth structure and patient preference for conservative treatment	Immunocompromised patients (relative)

* SIP—Symptomatic Irreversible Pulpitis; STP—Sensitive to Percussion; CST—cold sensibility test. # 24 h is not an absolute contraindication. A 24 h–48 h window period has a decreased success rate, but younger patients have a better prognosis.

**Table 2:** Participant demographics.

	Gender		Age		Tooth	
Case Diagnosis	Male	Female	Mean ± SD	Range	Molar	Premolar
Reversible Pulpitis	6	4	43.6 ± 15.5	23–71	8	2
Irreversible Pulpitis	11	6	36.1 ± 14.0	15–59	10	7
Total	17	10			18	9

**Table 3:** PRP post-operative pain reduction and correlation with pre-operative pain *.

Pre-Op Pain Severity (0–10 VAS)	Total Patients	Success in the Same Evening	Success at 24 Hours	Success at 48 Hours
Low (0–3)	8	8 (100%)	8 (100%)	8 (100%)
Moderate (4–6)	4	2 (50%)	4 (100%)	4 (100%)
High (7–10)	15	10 (66.7%)	14 (93.3%)	15 (100%)
Overall pain reduction success		20 (74.1%)	26 (96.3%)	27 (100%)

* Success in post-operative pain reduction is defined as post-operative pain rated as ≤1/10 VAS.

**Table 4:** Comparison of post-operative immediate pain reduction on the evening following treatment in different subgroups.

Subgroup	*n*	Mean Reduction *	Median Reduction *	Test	*p*-Value
Spontaneous Pain: Yes	17	5.68	6.5	Mann–Whitney U	<0.001
Spontaneous Pain: No	10	2.15	2		
Male	17	3.97	4	Mann–Whitney U	0.861
Female	10	5.15	5.25		
Age ≤ 38 years	13	5.29	6.75	Mann–Whitney U	0.019
Age > 38 years	14	3.27	2		

* Mean reduction and median reduction are post-op reduction from pre-op pain expressed as VAS score (1–10).

## Data Availability

The original contributions presented in this study are included in the article or Supplementary Material. Further inquiries can be directed to the corresponding author.

## Supplementary Materials

The following supporting information can be downloaded at: https://www.mdpi.com/article/10.3390/dj14090571/s1, Table S1: Patient data and treatment results. Figure S1: Varying volumes of liquid GA on a micro-applicator for single-tooth application. Figure S2: GA–RMGI solid exhibits strong bactericidal activity without direct contact. Table S2: Bactericidal activity of solid glutaraldehyde (GA) formed with different light-cured dental cavity liners/bases. Figure S3: Short, self-limiting bactericidal activity of GA–RMGI solid; Figure S4: Transient bactericidal activity of GA–RMGI solid.
