# Supplementary material for: A Conservative Restorative Protocol for Symptomatic Irreversible Pulpitis: Clinical Success and Rapid Pain Relief in a 27-Case Series with 36-Month Follow-Up
Source: Dent J (Basel). 2026 Sep 7;14(9):571. doi: 10.3390/dj14090571 (PMC13605355; doi:10.3390/dj14090571)
Supplement: Supplementary file 1 [file dentistry-14-00571-s001.zip › dentistry-4468982-supplementary.pdf]

Supplementary Materials

Table S1. Patient Data and Treatment Results *.

Case	Tooth #	Sex	Age	Pre-Op Pain (VAS)	Spontaneous Pain	STP	Pre-Op CST	Spontaneous Pain Duration	Post-Op Pain Same Evening	Post-Op Pain at 24h	Post-Op Pain at 48 h	Post-Op Radiograph	Post-Op CVT	Follow-Up Duration
1	26	M	17	6–7	Yes	Yes	Painful	<12 h	0	0	0	Atypical Apex change	Normal	25–36 Months
2	47	F	15	7	Yes	Yes	Painful	Few h	0	0	0	No change at Apex	Normal	25–36 Months
3	46	M	33	0–1	No	No	Sensitive	NA	0	0	0	No change at Apex	Normal	25–36 Months
4	16	F	39	2–3	No	No	Sensitive	NA	0	0	0	Atypical Apex change	Normal	25–36 Months
5	26	M	41	0–1	No	No	Sensitive	NA	0	0	0	No change at Apex	Negative	25–36 Months
6	14	M	32	8	Yes	Yes	Painful	12–24 h	0	0	0	No change at Apex	Normal	25–36 Months
7	15	F	38	8	Yes	Yes	Painful	1 h	0	0	0	Apical Lucency	Negative	13–24 Months
8	26	M	30	7	Yes	Yes	Painful	2 h	0	0	0	No change at Apex	Negative	13–24 Months
9	17	M	48	6–7	Yes	Yes	Painful	<12 h	0	0	0	No change at Apex	Normal	13–24 Months
10	14	F	24	2	No	No	Sensitive	NA	0	0	0	No change at Apex	Normal	13–24 Months
11	24	M	40	7	Yes	Yes	Painful	<24 h	4	2	0	No change at Apex	Normal	13–24 Months
12	38	F	54	0	No	No	Sensitive	NA	0	0	0	No change at Apex	Normal	13–24 Months
13	15	M	22	8	Yes	Yes	Painful	24–36 h	1	0	0	No change at Apex	Sensitive	13–24 Months
14	27	M	67	2	No	No	Sensitive	NA	1	0	0	No change at Apex	Normal	13–24 Months
15	17	F	57	8–9	Yes	Yes	Lingering pain	24 h	0	0	0	No change at Apex	Normal	13–24 Months
16	15	M	34	9	Yes	Yes	Pain	24h	5	1	0	No change at Apex	Sensitive	13–24 Months
17	26	F	40	2	No	No	Sensitive	NA	0	0	0	No change at Apex	Normal	13–24 Months
18	26	M	40	4	Yes	Yes	Lingering pain	Few h	0	0	0	No change at Apex	Sensitive	13–24 Months
19	28	F	59	6	Yes	Yes	Very Painful	Few h	4	0	0	No change at Apex	Normal	13–24 Months
20	15	M	23	5	No	Yes	Very Sensitive	NA	0	0	0	No change at Apex	Normal	13–24 Months
21	25	F	35	7	Yes	Yes	Painful	<24 h	0	0	0	No change at Apex	Normal	7–12 Months
22	48	M	48	2	No	No	Painful	NA	0	0	0	No change at Apex	Sensitive	7–12 Months
23	37	M	71	6	No	No	Normal	NA	2	0	0	No change at Apex	Normal	7–12 Months
24	34	M	47	7–8	Yes	Yes	Painful	24 h	4	0	0	No change at Apex	Normal	7–12 Months
25	46	M	17	8	Yes	Yes	Sensitive	24 h	4	0	0	No change at Apex	Normal	3–6 Months
26	27	M	34	7–8	Yes	Yes	Painful	48 h	0	0	0	No change at Apex	Normal	3–6 Months
27	17	F	52	8	Yes	Yes	Painful	< or =24 h	2	0	0	No change at Apex	Sensitive	3–6 Months

* Tooth# (FDI system, ISO 3950); VAS (Visual Analog Scale); Pre-op (Pre-operative); Post-op(Post-operative); STP (Sensitivity to Percussion); CST (Cold Sensibility Test).

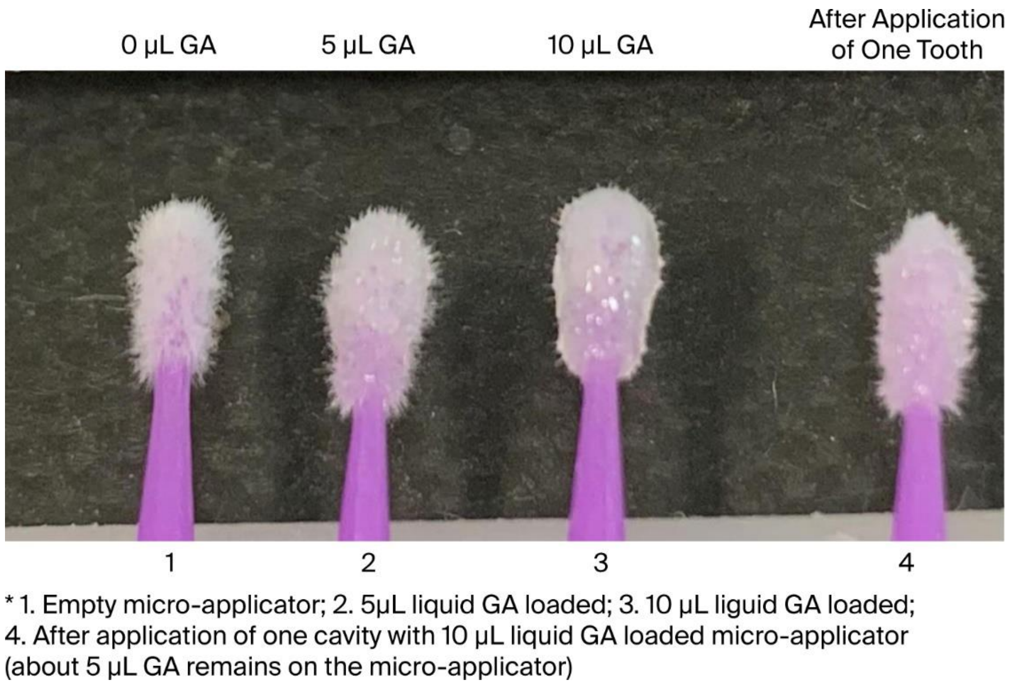

Figure S1. Varying Volumes of Liquid GA on a Micro-applicator for Single-tooth Application *.

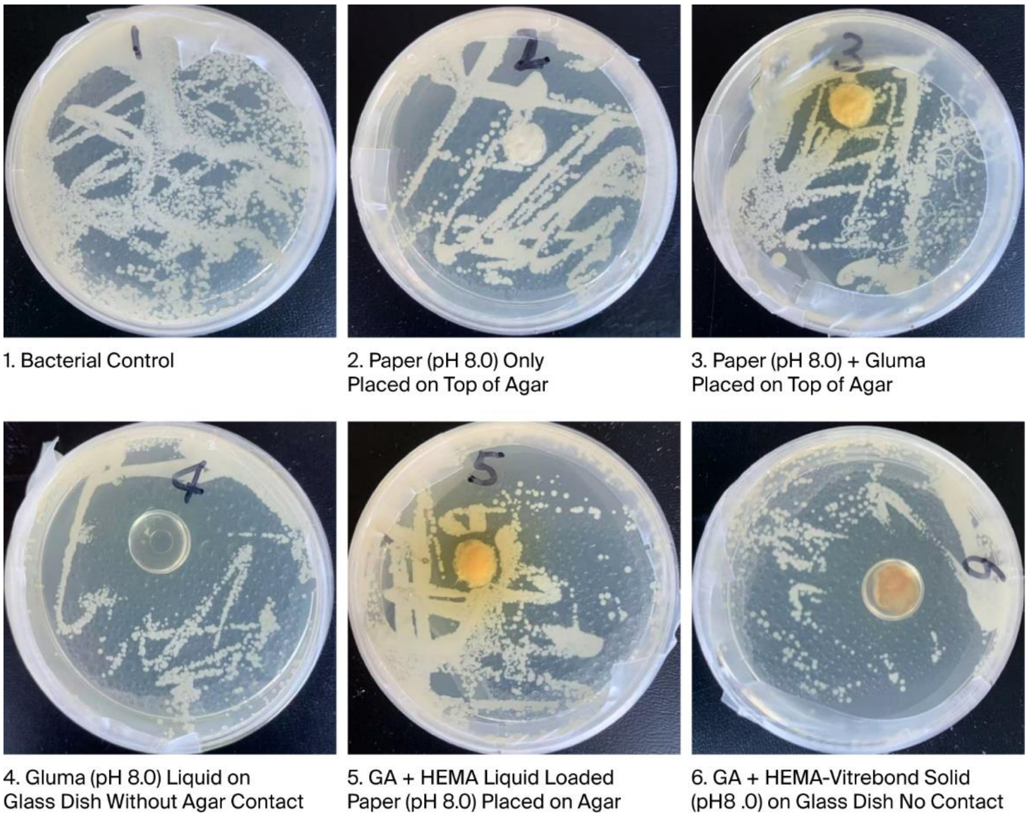

FigureS2. GA–RMGI solid exhibits strong bactericidal activity without direct contact. Tryptic soy agar plates were inoculated with oral bacterial culture suspension with a 10 μL-inoculation metal loop. For direct-contact experiments, 10 μL of liquid GA/HEMA or Gluma Desensitizer loaded paper discs were placed directly on the agar surface. For non-contact (vapor-phase) experiments, GA-containing liquids or solids in glass dishes positioned at the bottom of inverted Petri dishes; the agar plates were placed face down above them so that they shared the same enclosed head-space without touching the agar surface. The Petri dishes were tightly sealed with Parafilm and

incubated at 37 °C for 24 h. The presence of bacterial growth inhibition halos and radial width (mm) of inhibition zones surrounding the discs was measured, and qualitative changes in colony density across the entire plate were recorded. The results show that paper-only control (Petri dish 2) was the same as the bacterial control (Petri dish 1) and had no bacterial inhibition effect. Both Gluma solution-loaded absorbent paper (Petri dish 3) and GA+HEMA-loaded paper (Petri dish 5) directly contacting the agar have a minimal bactericide effect with less than a 1 mm inhibition zone. Liquid Gluma solution in a glass dish (Petri dish 4) without agar contact has a large non-bacterial growth zone of 7.5 mm (from the glass disc edge to the zone edge). Solid Vitrebond + GA (Petri dish 6) without agar contact also has a large inhibition zone of 6 mm. These findings indicate that GA, whether in liquid or solid form, can exert potent bactericidal activity via vapour-phase diffusion when not consumed by immediate reaction with proteins in the agar. Conversely, direct contact between GA and the protein-rich agar appeared to quench GA activity rapidly, consistent with extensive crosslinking and depletion of reactive aldehyde groups.

Table S2. Bactericidal activity of solid glutaraldehyde (GA) formedwith different light-cured dental cavity liners/bases*.

Material Combination	Bactericidal Activity
Gluma liquid	Positive
GA + HEMA liquid	Positive
Vitrebond Plus-Gluma solid	Positive
Vitrebond Plus-GA + HEMA solid	Positive
Activa-Gluma solid	Positive
Vitrebond Plus solid (no GA)	Negative
Lime-Lite Enhanced solid (no GA)	Negative
Lime-Lite Enhanced-Gluma solid	Negative
Activa solid (no GA)	Negative
TheraCal LC solid (no GA)	Negative
Thera Cal LC-Gluma solid	Negative

*No liner/base material alone (without GA) exhibited any bactericidal activity under the tested conditions. Among GA-containing solids, only combinations containing Vitrebond Plus or ACTIVA™ BioACTIVE-BASE/LINER™ produced detectable inhibition zones and reduction in colony density. GA-containing solids prepared with Lime-Lite Enhanced or TheraCal LC showed no observable bactericidal effect, suggesting that the physicochemical properties of the liner matrix are critical for GA vapor release.

Material

- GA +HEMA solution (6.25% Glutaraldehyde solution containing 37.5% HEMA);
- GLUMA Desensitizer; (Kulzer, Wehrheim, Germany);
- Vitrebond Plus (3M U.S.A.);
- Lime-Lite™ Enhanced, Light Cure Cavity Liner (Pulpdent U.S.A.);
- ACTIVA™ BioACTIVE-BASE/LINER™ (Pulpdent U.S.A.);
- TheraCal LC (BISCO, U.S.A.).

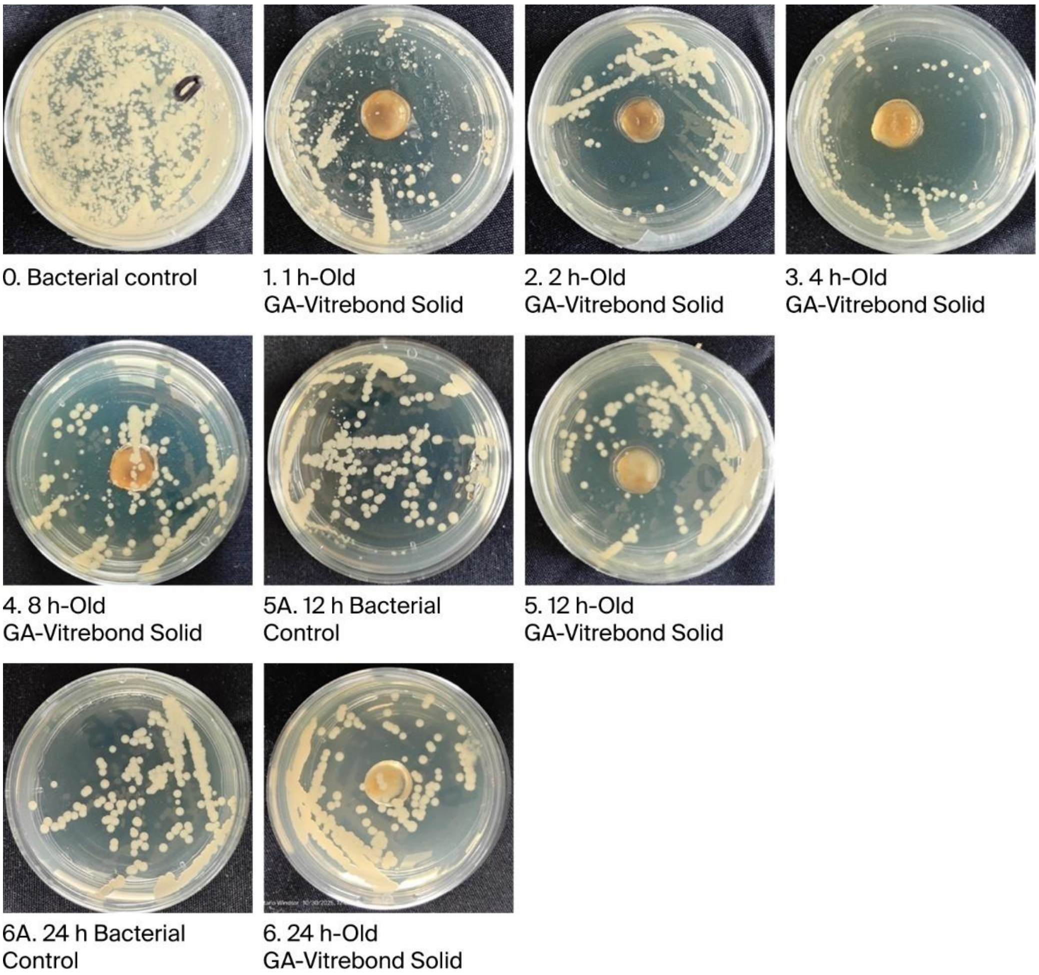

Figure S3. Short, self-limiting bactericidal activity of GA–RMGI solid. To evaluate the time kinetics of GA-RMGI solid bactericidal activity, identical GA–RMGI solid discs were prepared using 10 μL GA and mixed with Vitrebond Plus on each small glass dishes and solidified under curing light. These GA-RMGI solid discs were stored at 37 °C and tested in the bacterial inhibition assay at predetermined time points (1h, 2h, 4 h, 8 h, 12 h, 24 h and a week after formation) in sealed agar Petri dishes under non-contact conditions. Bacterial colonies were counted and compared to the numbers on bacterial control agar dishes and the reductions were expressed as a percentage. Three same test numbers were combined and average numbers were used.

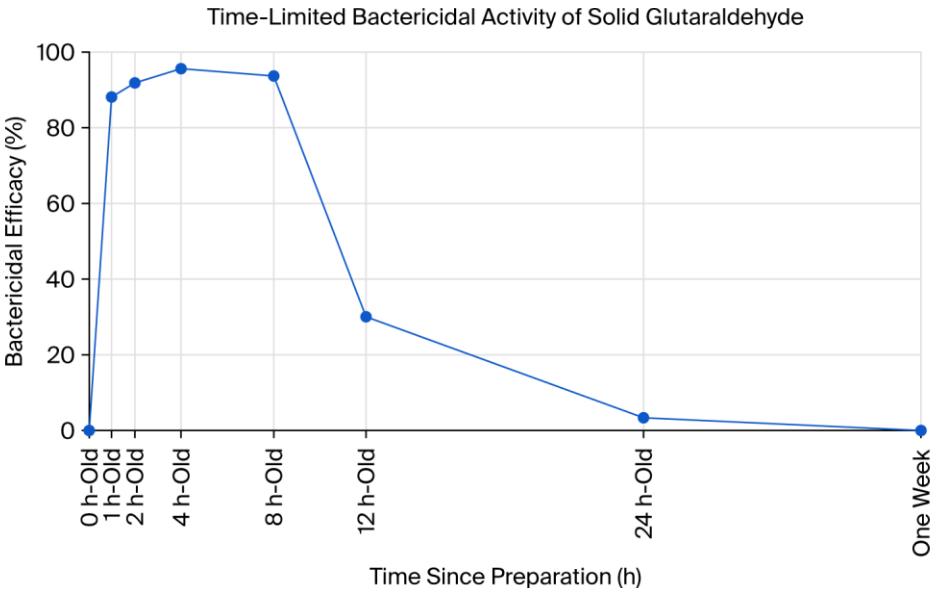

FigureS4. Transient bactericidal activity of GA–RMGI solid. To evaluate the time kinetics of GA-RMGI solid activity, identical GA–RMGI solid discs were prepared using 10 μL GA and mixed with Vitrebond Plus on each small glass dishes and solidified under curing light. These GA-RMGI solid discs were stored at 37 °C and tested in the bacterial inhibition assay at predetermined time points (1h, 2h, 4 h, 8 h, 12 h, 24 h and a week after formation) in sealed agar Petri dishes under non-contact conditions. Bacterial colonies were counted and compared to the numbers on bacterial control agar dishes and the reductions of bacterial colony (bactericidal efficacy) were expressed as a percentage. Three same test numbers were combined and average numbers were used.
